# Grape Seed Proanthocyanidins Inhibit Migration and Invasion of Bladder Cancer Cells by Reversing EMT through Suppression of TGF-*β* Signaling Pathway

**DOI:** 10.1155/2021/5564312

**Published:** 2021-07-27

**Authors:** Ninggang Yang, Jing Gao, Ruizhen Hou, Xiaoli Xu, Ningqiang Yang, Shuangsheng Huang

**Affiliations:** ^1^Department of Urology, The First People's Hospital of Lanzhou City, Lanzhou 730050, China; ^2^Hospital of Northwest Minzu University, Lanzhou 730030, China; ^3^The First School of Clinical Medicine, Lanzhou University, Lanzhou 730000, China; ^4^School of Basic Medicine, Lanzhou University, Lanzhou 730000, China; ^5^Department of Urology, Lanzhou University Second Hospital, Lanzhou 730030, China; ^6^Medical Department of Northwest Minzu University, Lanzhou 730030, China

## Abstract

Bladder cancer (BC) is the most common cancer of the urinary system. Despite advances in diagnosis and therapy, the prognosis is still poor because of recurrence and metastasis. Epithelial-mesenchymal transition (EMT) is considered to play an important role in the invasion and metastasis of BC. Grape seed proanthocyanidins (GSPs) exhibit chemopreventive and chemotherapeutic activities against several types of cancer. However, their effects and underlying mechanisms on the invasive potential of BC remain unclear. In this study, we found that GSPs inhibited migration, invasion, and MMP-2/-9 secretion of both T24 and 5637 bladder cancer cells at noncytotoxic concentrations. We also discovered that 5637 cells were more suitable than T24 cells for the EMT study. Further study showed that GSPs inhibited EMT by reversing the TGF-*β*-induced morphological change and upregulation of mesenchymal markers N-cadherin, vimentin, and Slug as well as downregulation of epithelial markers E-cadherin and ZO-1 in 5637 cells. GSPs also inhibited TGF-*β*-induced phosphorylation of Smad2/3, Akt, Erk, and p38 in 5637 cells without affecting the expression of total Smad2/3, Akt, Erk, and p38. Taken together, the results of the present study demonstrate that GSPs effectively inhibit the migration and invasion of BC cells by reversing EMT through suppression of the TGF-*β* signaling pathway, which indicates that GSPs could be developed as a potential chemopreventive and therapeutic agent against bladder cancer.

## 1. Introduction

Bladder cancer (BC) is the most common cancer of the urinary system. It ranks tenth among 36 cancers worldwide, with an estimated 549,000 new cases and about 200,000 deaths during 2018. Especially in men, it is the sixth most common cancer and the ninth leading cause of cancer death [1]. In China, BC is the 13th most common cancer and the 12th leading cause of cancer death. It is estimated that there were 78,100 new cases and 32,100 cancer death in 2014 [2]. Furthermore, according to Cai et al., both incidence and mortality of BC will continue to increase among some countries and regions in the next 15 years [3]. Despite significant progresses in combined therapies including surgery, chemotherapy, and radiotherapy, BC is still characterized by a poor prognosis and a low survival rate during the past decades, particularly as a result of recurrence and metastasis [4]. Therefore, it is necessary to identify mechanisms underlying BC development as well as develop novel therapeutic reagents for its treatment.

Epithelial-mesenchymal transition (EMT) is a process in which polarized epithelial cells undergo a phenotypic transition that enables them to obtain mesenchymal cells traits. Hallmarks of EMT include the loss of expression of epithelial markers, such as E-cadherin and occludins, and the concomitant increase of mesenchymal markers including N-cadherin and vimentin [5–7]. EMT is considered a pathological process that promotes cancer progression, particularly invasion and metastasis. Through this process, cancer cells detach from the primary tumor and acquire migratory and invasive capabilities as well as resistance to therapy [8–10]. Therefore, inhibition of EMT would be an effective measure to block cancer invasion and metastasis. The most well-characterized factor responsible for the induction of EMT by far is TGF-*β*. TGF-*β* has a dual role in tumor progression. In the early stage, TGF-*β* is considered as a tumor suppressor by inducing cell differentiation and apoptosis. In the late stage, TGF-*β* contributes to cancer progression by promoting tumor invasion and metastasis, which is related to its induction of EMT [11, 12]. In bladder cancer, studies have shown that cancer-associated fibroblasts induce bladder cancer cell EMT and invasiveness through secreting TGF-*β* [13]. Therefore, inhibition or reversion of the TGF-*β* signaling pathway-mediated EMT might be an effective measure for bladder cancer treatment.

It is reported that many polyphenolic compounds have anti-EMT activity [14, 15]. Proanthocyanidins are dimers, trimers, and other oligomers of flavan-3-ols. As a kind of natural polyphenolic compounds, proanthocyanidins have significant antioxidant activities and are rich in grapes, especially in grape seeds [16]. Grape seed proanthocyanidins (GSPs) have been reported to possess chemopreventive and chemotherapeutic potential against several types of cancers. The underlying mechanisms are related to inhibition of proliferation, induction of apoptosis, arrest of cell cycle, inhibition of angiogenesis, and suppression of metastasis [17–19]. GSPs have also been shown to have the ability to reverse the process of EMT [20–22]. However, the effects and underlying mechanisms of GSPs on the invasive potential of BC remain unclear.

In the present study, we investigated the antimetastic activity and associated mechanisms of GSPs against human BC cells *in vitro*. Our results show that GSPs inhibit the migration, invasion, and MMP-2/9 secretion of both T24 and 5637 bladder cancer cells. GSPs also inhibit TGF-*β*-induced EMT of 5637 cells, which are related to the blocking of the TGF-*β* signaling pathway. Hence, the present study provides scientific evidences supporting GSPs as chemopreventive and chemotherapeutic agents against BC.

## 2. Materials and Methods

### 2.1. Chemicals and Reagents

Grape seed proanthocyanidins (GSPs) containing ≥95% proanthocyanidins, ≥1.8% proanthocyanidins B2, and ≥60% oligomers were obtained from Tianjin Jianfeng Natural Product R&D Co., Ltd. (Tianjin, China). RPMI-1640 culture medium was obtained from Gibco (Thermo Fisher Scientific, Inc., Waltham, MA, USA). Fetal bovine serum (FBS) was purchased from National HyClone (Lanzhou) Bio-engineering Co., Ltd. (Lanzhou, China). Sulforhodamine B (SRB) and gelatin from porcine skin were obtained from Sigma-Aldrich (Merck KGaA, Darmstadt, Germany). Millicell Cell Culture Inserts were obtained from EMD Millipore (Billerica, MA, USA). Matrigel and transforming growth factor-*β* (TGF-*β*) was purchased from BD Biosciences (Franklin Lakes, NJ, USA). RIPA lysis buffer (protease and phosphatase inhibitors added) was purchased from Beyotime Institute of Biotechnology (Shanghai, China). Primary antibodies directed against N-cadherin, vimentin, E-cadherin, and *β*-actin were purchased from Abcam (Cambridge, MA, USA). Primary antibodies directed against ZO-1, Slug, Smad 2/3, p-Smad2 (Ser465/467)/Smad3 (Ser423/425), Akt, p-Akt, p44/42 MAPK (Erk1/2), p-p44/42 MAPK (Erk1/2), p38 MAPK, p-p38 MAPK, and horseradish peroxidase-conjugated goat anti-rabbit IgG were purchased from Cell Signaling Technology (Danvers, MA, USA). SuperSignal™ West Pico PLUS Chemiluminescent Substrate was purchased from Thermo Scientific (Waltham, MA, USA). GSPs were dissolved in DMSO as stock and diluted to appropriate concentrations with RPMI 1640 medium before use. The maximum final concentration of DMSO in cultures was less than 0.5%.

### 2.2. Cell Culture

Human bladder cancer cell lines T24 and 5637 were obtained from the Type Culture Collection of the Chinese Academy of Sciences (Shanghai, China) and cultured in RPMI 1640 medium supplemented with 10% FBS at 37°C and 5% CO_2_.

### 2.3. Cell Viability Assay

The viability of cells was determined by the SRB assay as previously described [23] with certain modifications. Cells were plated into 96-well plates at a density of 4 − 6 × 10^3^ cells/well in 100 *μ*l 1640 medium supplemented with 10% FBS. Following incubation at 37°C overnight, cells were treated with GSPs at varying concentrations (0-200 *μ*g/ml) for 24, 48, or 72 h. Subsequently, the cultures were fixed with cold 10% trichloroacetic acid at 4°C for 1 h and washed with water. Next, the plates were air-dried and the fixed cells were stained with 0.4% SRB at room temperature for 10 min and washed repeatedly with 0.1% acetic acid to remove the unbound dye. The bound SRB was dissolved in 1% Tris (pH 10.5). The optical density was measured at 515 nm using a microplate reader.

### 2.4. Cell Migration Assay

Cell migration was assayed in Millicell Cell Culture Inserts using a polycarbonate filter with a pore size of 8 *μ*m as previously described [24]. Cells were trypsinized and suspended in a serum-free 1640 medium. A total of 8 × 10^4^ cells in 0.4 ml medium were seeded into the upper chamber of cell culture inserts with various concentrations of GSPs (0-50 *μ*g/ml). The culture inserts were then placed into 24-well plates filled with 0.6 ml 1640 medium supplemented with 20% FBS as a chemoattractant. Following incubation at 37°C for 24 h, the nonmigrated cells on the upper surface of the membrane were wiped off with a cotton swab. Cells that had crossed over the inserts were fixed with methanol for 30 min and then stained with 0.1% crystal violet for 10 min at room temperature. Images were captured using light microscopy (magnification, ×100). The number of cells that had migrated was counted using the Image J software (version 1.52v; National Institutes of Health, Bethesda, MD, USA).

### 2.5. Cell Invasion Assay

A cell invasion assay was performed as previously described [25]. Briefly, the upper chamber of Millicell Cell Culture Inserts using a polycarbonate filter with a pore size of 8 *μ*m was coated with 50 *μ*l Matrigel diluted 1 : 8 with PBS. Subsequently, 4 × 10^5^ cells in 0.4 ml serum-free 1640 medium with or without GSPs (0-50 *μ*g/ml) were added to the upper chamber. The culture inserts were then placed into 24-well plates. Subsequently, the lower chamber was filled with 0.6 ml 1640 medium supplemented with 20% FBS as a chemoattractant to induce invasion. Following incubation at 37°C for 24 h, the culture inserts were removed, and the noninvasive cells on the upper surface of culture inserts were scraped away with a cotton swab. The cells that invaded through the Matrigel were fixed with methanol for 30 min and then stained with 0.1% crystal violet for 10 min at room temperature. Images were captured by light microscopy (magnification, ×100), and the number of cells was counted using the Image J software.

### 2.6. Gelatin Zymography

The enzymatic activities of MMP-2 and MMP-9 were examined by gelatin zymography as described previously [24]. Subconfluent cells were treated with GSPs (0-50 *μ*g/ml) for 24 h in a serum-free 1640 medium. Following treatment, the conditioned medium was collected and centrifuged at 300 × g for 10 min at 4°C to remove cellular debris. The supernatants were incubated with sample buffer (without *β*-mercaptoethanol) for 0.5 h at 37°C and then subjected to 7.5% SDS-PAGE in a gel containing 1% gelatin. Following electrophoresis, the gels were washed twice with washing buffer [50 mM Tris-HCl (pH 7.5), 100 mM NaCl and 2.5% Triton X-100] for 1 h and incubated in a reaction buffer [50 mM Tris-HCl (pH 7.5), 150 mM NaCl and 10 mM CaCl_2_] at 37°C for 36 h. Subsequently, the gels were stained with 0.25% Coomassie Blue R250 for 30 min and destained for 10 min with 25% methanol and 7.5% acetic acid at room temperature. Enzyme activity was visualized as a bright band on a blue background. The band intensities were measured using the Adobe Photoshop CS5 software.

### 2.7. Western Blot Analysis

After treatment with TGF-*β* or TGF-*β* with or without GSPs for 24 h, cells were lysed using RIPA buffer. Total protein was collected and then quantified using the Bradford assay. Protein was separated via 10% SDS-PAGE (40 *μ*g protein/lane). Following electrophoresis, the separated proteins were transferred to PVDF membranes. Next, the membranes were probed with the primary antibodies at 4°C overnight. Following this, the membranes were incubated with horseradish peroxidase-conjugated secondary antibodies at 4°C for 2 h. The desired proteins were detected using SuperSignal West Pico Chemiluminescent Substrate according to the manufacturer's protocol. Bands were quantified using Adobe Photoshop CS5.

### 2.8. Statistical Analysis

Data were expressed as the mean ± SD. Statistical significance was determined by Student's *t*-test and ANOVA using the SPSS software (version 20; SPSS, Inc., Chicago, IL, USA). *P* < 0.05 was considered statistically significant.

## 3. Results

### 3.1. GSPs Decrease the Viability of BC Cells

**Figure 1:**
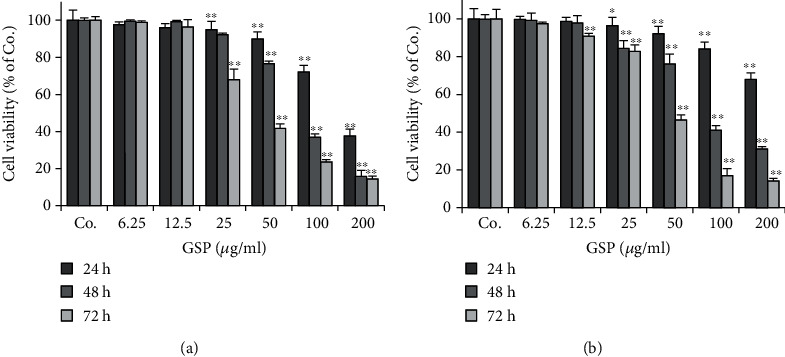
GSPs decrease cell viability of T24 (a) and 5637 (b) cells. After being treated with GSPs for the indicated time, the cell viability was measured with the SRB assay. The data were presented as mean ± SD (*n* = 6). ^∗^*P* < 0.05, ^∗∗^*P* < 0.01 vs. control group.

The effects of GSPs on the viability of BC cells were examined using the SRB assay. As shown in Figure 1, treatment with different concentrations of GSPs in different times inhibited the viability of both T24 and 5637 cells in a dose-dependent manner. The half-maximal inhibitory concentration values (IC_50_) to T24 cells were 206.8, 86.9, and 56.2 *μ*g/ml for 24, 48, and 72 h. Accordingly, the IC_50_ values to 5637 cells for 24, 48, and 72 h were 288.3, 98.0, and 52.6 *μ*g/ml, respectively. As treatment with 6.25-50 *μ*g/ml GSPs for 24 h had no significant inhibitory effects on the viability of both T24 and 5637 cells, we carry out subsequent experiments under these conditions.

### 3.2. GSPs Inhibit Migration of BC Cells

**Figure 2:**
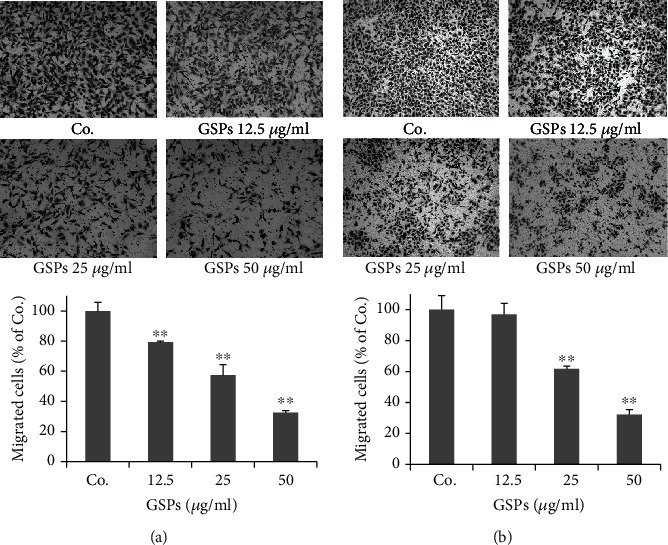
GSPs inhibit migration of T24 (a) and 5637 (b) cells. After incubated with GSPs for 24 h in the upper chamber of Millicells, cells passed through the membrane were fixed, stained, and counted. Representative images were shown. The data were presented as mean ± SD (*n* = 4). ^∗∗^*P* < 0.01 vs. control group.

Cancer cell migration and invasion are the initial and critical steps in the process of cancer metastasis. We then investigated the effects of GSPs on the migration of BC cells using a transwell migration assay. After stimulated T24 cells with 20% FBS for 24 h, a large number of cells migrated to the lower side of the chamber. Treatment with different concentrations of GSPs inhibited the FBS-induced migration of T24 cells in a dose-dependent manner. The inhibition rates of 12.5, 25, 50 *μ*g/ml GSPs were 20.7%, 42.6%, and 67.5%, respectively (Figure 2(a)). The similar inhibitory effects of GSPs were also observed on 5637 cells. As shown in Figure 2(b), GSPs treatments inhibited migration of 5637 cells dose-dependently with the inhibition rates of 3.1%, 38.2%, and 67.9% for 12.5, 25, and 50 *μ*g/ml GSPs, respectively.

### 3.3. GSPs Inhibit Invasion of BC Cells

**Figure 3:**
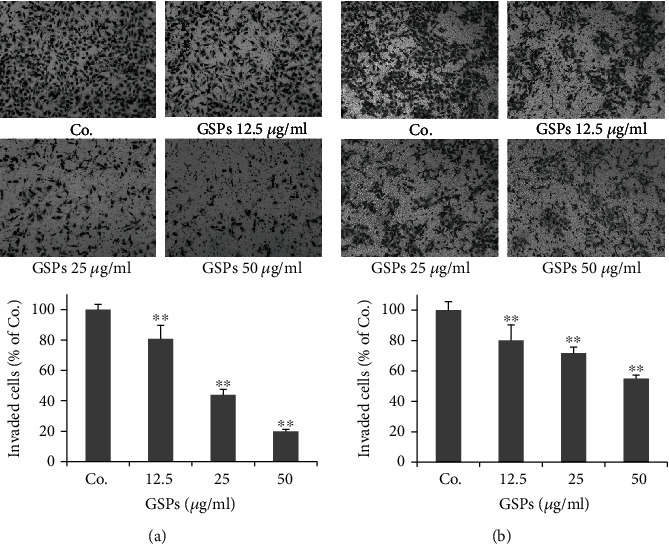
GSPs inhibit invasion of T24 (a) and 5637 (b) cells. After incubated with GSPs for 24 h in the upper chamber of Millicells, cells passed through the membrane coated with Matrigel were fixed, stained, and counted. Representative images were shown. The data were presented as mean ± SD (*n* = 4). ^∗∗^*P* < 0.01 vs. control group.

The effects of GSPs on the invasive capacity of BC cells were also investigated. As shown in Figure 3(a), the addition of 20% FBS in the lower chamber significantly induced the invasion of T24 cells. GSPs significantly inhibited FBS-induced invasion of T24 cells dose-dependently. The inhibition rates of GSPs at 12.5, 25, and 50 *μ*g/ml were 19.1%, 56.0%, and 80.0%, respectively. Similarly, GSPs treatment for 24 h also inhibited the invasion of 5637 cells. The inhibition rates of GSPs at 12.5, 25, and 50 *μ*g/ml were 19.8%, 28.2%, and 45.1%, respectively (Figure 3(b)).

### 3.4. GSPs Inhibit the Secretion of MMP-2 and MMP-9 by BC Cells

**Figure 4:**
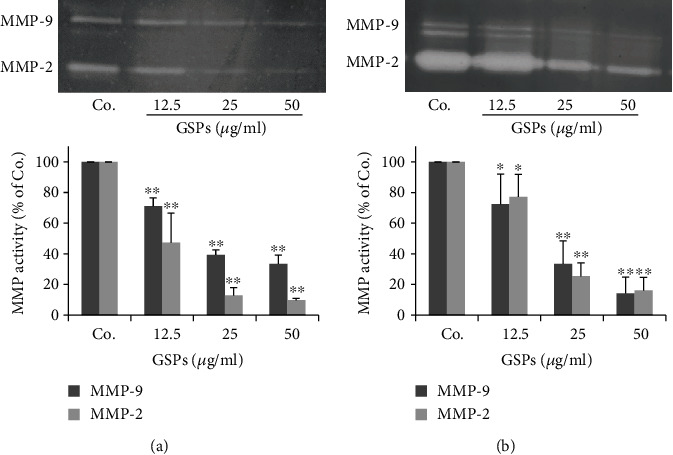
GSPs inhibit the secretion of MMP-2/9 of T24 (a) and 5637 (b) cells. Subconfluent cells were treated with different concentrations of GSPs in a serum-free medium for 24 h. The conditioned medium was collected. The activities of MMP-2 and MMP-9 in conditioned medium were detected by gelatin zymography and the results were quantified. The data were presented as mean ± SD (*n* = 3). ^∗^*P* < 0.05, ^∗∗^*P* < 0.01 vs. control group.

The secretion of MMPs for extracellular matrix degradation is a key event in the process of tumor metastasis. So we further examined the effects of GSPs on the secretion of MMP-2 and MMP-9 by BC cells. Gelatin zymography showed that MMP-2 and MMP-9 activities in the conditioned medium collected from both T24 and 5637 cells were markedly reduced by GSPs dose-dependently (Figure 4).

### 3.5. Establishment of TGF-*β*-Induced EMT Model in BC Cells

**Figure 5:**
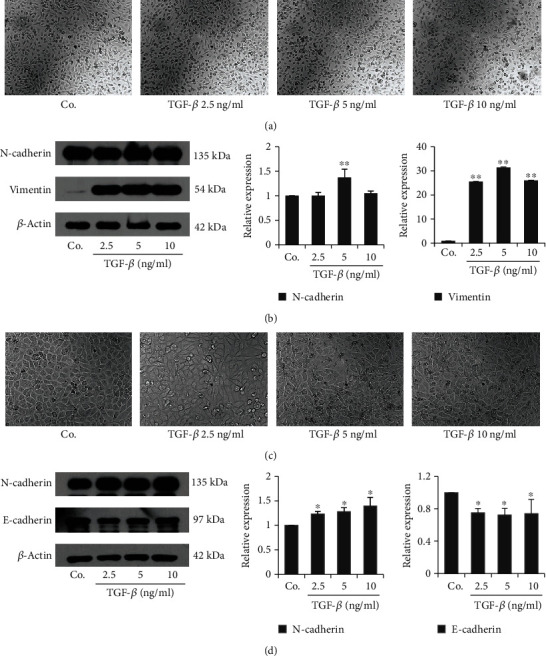
Establishment of TGF-*β*-induced EMT model of BC cells. T24 (a, b) and 5637 (c, d) were treated with TGF-*β* for 24 h. The morphological changes were observed under a phase-contrast microscope and photographed (100x). The level of EMT markers was examined by western blotting and the results were quantified. The data were presented as mean ± SD (*n* = 3). ^∗^*P* < 0.05, ^∗∗^*P* < 0.01 vs. control group.

EMT plays an important role in promoting tumor invasion and metastasis. TGF-*β* is one important inducer of EMT. To investigate whether GSPs decreased cancer migration and invasion through inhibiting EMT, we established the TGF-*β*-induced EMT model with T24 and 5637 cells. For this purpose, both T24 and 5637 cells were treated with different concentrations of TGF-*β* for 24 h and then cell morphology and EMT markers were detected. As shown in Figure 5, treatment with TGF-*β* for 24 h did not induce obvious morphologic changes of T24 cells (Figure 5(a)). Western blotting results showed that TGF-*β* treatment for 24 h increased the expression of mesenchymal biomarker vimentin in T24 cells. However, only 5 ng/ml TGF-*β* enhanced the expression of another mesenchymal biomarker N-cadherin (Figure 5(b)). We did not detect the expression of epithelial biomarker E-cadherin (data not shown) in T24 cells. In 5637 cells, treatment with different concentrations of TGF-*β* for 24 h resulted in a morphological change from the initial polygonal epithelial appearance to an elongated mesenchymal morphology (Figure 5(c)). The expression of the mesenchymal marker N-cadherin was increased, while the epithelial marker E-cadherin was decreased by TGF-*β* treatment (Figure 5(d)). These results indicated that 5637 cells were more suitable than T24 cells for the EMT study. Therefore, we selected 5637 cells and 5 ng/ml TGF-*β* treatment to establish the EMT model induced by TGF-*β*.

### 3.6. GSPs Inhibit TGF-*β*-Induced EMT in 5637 Cells

**Figure 6:**
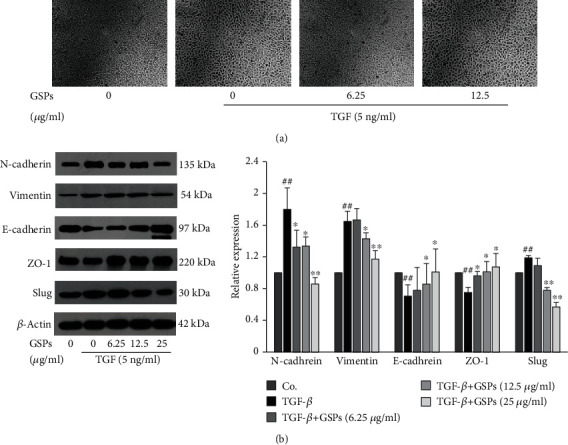
GSPs inhibit TGF-*β*-induced EMT in 5637 cells. 5637 cells were treated with GSPs in the presence or absence of 5 ng/ml of TGF-*β* for 24 h. (a) The morphological changes were observed under a phase-contrast microscope and photographed (100x). (b) The expression of EMT markers was detected by western blotting and the results were quantified. The data were presented as mean ± SD (*n* = 3). ^##^*P* < 0.01 vs. control group and ^∗^*P* < 0.05, ^∗∗^*P* < 0.01 vs. TGF-*β* treatment alone.

Consistent with results of Figure 5, treatment with TGF-*β* at the concentration of 5 ng/ml for 24 h led to a morphological change from an epithelial morphology to a mesenchymal phenotype in 5637 cells. However, GSPs reversed this morphological change induced by TGF-*β* (Figure 6(a)). Results from western blotting also showed that treatment with TGF-*β* at the concentration of 5 ng/ml for 24 h upregulated the expression of mesenchymal markers N-cadherin and vimentin as well as EMT-related transcription factors Slug but downregulated the expression of epithelial marker E-cadherin and ZO-1 in 5637 cells. GSPs treatment reversed the TGF-*β*-induced upregulation of N-cadherin, vimentin, and Slug as well as downregulation of E-cadherin and ZO-1 dose-dependently (Figure 6(b)). These results suggested that GSPs were able to inhibit TGF-*β*-induced EMT.

### 3.7. GSPs Inhibit TGF-*β* Signal Pathway

**Figure 7:**
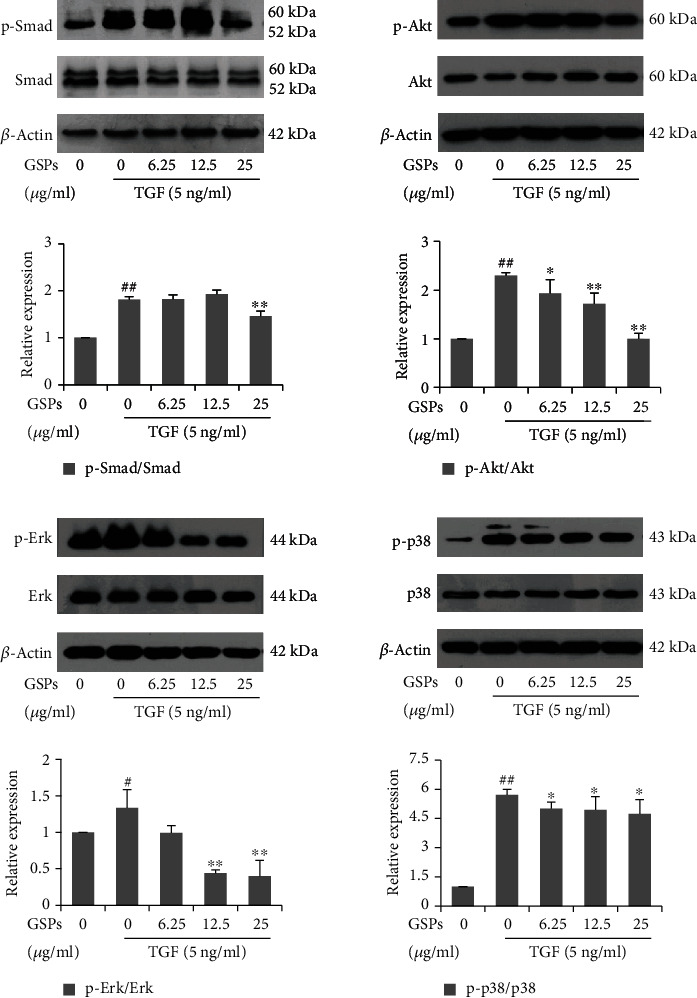
GSPs inhibit the TGF-*β* signal pathway. 5637 cells were treated with GSPs in the presence or absence of 5 ng/ml of TGF-*β* for 24 h. The expression of indicated signaling molecules was detected by western blotting and the results were quantified. The data were presented as mean ± SD (*n* = 3). ^#^*P* < 0.05, ^##^*P* < 0.01 vs. control group and ^∗^*P* < 0.05, ^∗∗^*P* < 0.01 vs. TGF-*β* treatment alone.

To elucidate potential molecular mechanisms of GSPs on EMT in 5637 cells, we examined the effects of GSPs on the activation of downstream signal molecules of TGF-*β*. As shown in Figure 7, treatment with TGF-*β* (5 ng/ml) enhanced the phosphorylation of Smad 2/3, Akt, Erk, and p38. GSPs treatment blocked the TGF-*β*-induced phosphorylation of Smad 2/3, Akt, Erk, and p38 without affecting the total SMAD2/3, Akt, Erk, and p-38 expression level.

## 4. Discussion

Increasing evidences demonstrated that many natural polyphenolic compounds including resveratrol and curcumin possessed a wide spectrum of pharmacological properties, including antimetastasis activities. The mechanisms of this action were mainly related to the inhibition of EMT phenotype through suppression of multiple signaling pathways, such as PI-3K/Akt, NF-*κ*B, FAK, and TGF-*β*1/SMADs pathway [26–29]. GSPs have also been shown to inhibit the metastasis of various types of cancer including tongue squamous cell carcinoma [30], nonsmall cell lung cancer [31], pancreatic cancer [21], and lung cancer [32]. The molecular targets of GSPs include NF-*κ*B, PI3K/Akt, and MAPK signaling pathway. The goal of this study was to investigate the antimetastatic activity and associated mechanisms of GSPs against human BC cells *in vitro*. We found that GSPs inhibited the migration, invasion, and secretion of MMP-2/9 of T24 and 5637 cells at noncytotoxic concentrations.

EMT has an important role in promoting the invasion and metastasis of cancer cells. This process is regulated by multiple signaling molecules. Among them, TGF-*β* is a potent stimulator of EMT [33]. Therefore, we select TGF-*β* to establish the EMT model of bladder cancer cells. Although Both T24 and 5637 cells are derived from bladder cancer, we found that morphology change induced by TGF-*β* was more obvious in 5637 cells than in T24 cells. Moreover, in T24 cells, TGF-*β* treatment only increased the expression of the mesenchymal marker N-cadherin and vimentin. No expression of epithelial marker E-cadherin was detected. Unlike T24 cells, treatment of 5637 cells with TGF-*β* resulted in the increased expression of N-cadherin, at the same time, the reduced expression of E-cadherin. The results were consistent with previous studies [34, 35]. Further studies showed that treatment 5637 cells with TGF-*β* for 24 h led to the increased expression of N-cadherin, vimentin, and Slug and decreased of E-cadherin and ZO-1, indicating the occurrence of EMT. All the results indicated that 5637 cells were better suited for EMT study than T24 cells. Therefore, we selected 5637 to establish the TGF-*β*-induced EMT model.

It has been reported that GSPs inhibit EMT in head and neck cutaneous squamous cell carcinoma cells and pancreatic cancer cells [20, 21]. However, whether GSPs can also inhibit EMT in BC cells remains to be explored. In the present study, we found that treatment with GSPs reversed the TGF-*β*-induced morphological changes as well as the expression of EMT markers. These suggest that GSPs have the ability to inhibit EMT in 5637 cells and that this may be one of the possible mechanisms through which GSPs inhibit the migration and invasion of BC cells.

It is reported that TGF-*β* induces EMT through a Smad-dependent canonical pathway and the Smad-independent noncanonical pathway. In the Smad-dependent pathway, the binding of TGF-*β* to cell surface receptor results the phosphorylation and activation of Smad2 and/or Smad3. The receptor-activated Smad2 and/or Smad3 then form Smad complexes with Smad4 and translocate into the nucleus to regulate transcription of target genes that mediate EMT. In addition to the Smad signaling pathways, TGF-*β* also induces EMT in certain types of cells through Smad-independent, noncanonical pathway, which involves the activation of other signaling pathways such as RHO-like GTPases, PI3K-Akt, and MAPK (including ERK, p38, and JNK) [36–38]. Therefore, many natural compounds were reported to inhibit EMT through targeting TGF-*β* signaling. Lee et al. reported that Farnesol, a fragrant component of essential oils, inhibited TGF-*β*-induced EMT through blocking the PI3K/Akt/mTOR signaling pathway in lung cancer cells [39]. Bergamottin, a naturally occurring furanocoumarin, suppressed the metastasis of lung cancer cells through inhibiting the TGF-mediated EMT process by blocking the activation of PI3K/Akt/mTOR signaling [40]. In the present study, treatment with TGF-*β* induced the phosphorylation of Smad2/3, Akt, Erk, and p38 in 5637 cells, suggesting that both Smad-dependent and non-Smad-dependent signaling pathways are related to TGF-*β* induced EMT. GSPs blocked the TGF-*β*-induced phosphorylation of Smad2/3, Akt, Erk, and p38 in 5637 cells but did not affect the total expression levels of these signal molecules, indicating that the reverse of TGF-*β*-induced EMT by GSPs is associated with the suppression of the TGF-*β* signaling pathway.

However, it is important to note that although our results have demonstrated that GSPs inhibit EMT through suppression of the TGF-*β* signaling pathway. It would make the results more convincing to further explore the mechanisms of GSPs by blocking or overexpressing the expression of downstream effectors of TGF-*β* signaling, such as Smad, Akt, p38 MAPK, and Erk, using siRNAs. Moreover, many studies have shown that in addition to TGF-*β*, EMT can be initiated by a variety of signaling molecules, including microRNA [41, 42]. It is necessary to establish EMT models induced by other signaling molecules to explore the effects and mechanisms of GSPs. In addition, this is a preliminary study on cell culture. Other BC-derived cell lines and *in vivo* studies will be needed in the future to further confirm the findings.

## 5. Conclusions

In conclusion, the results of our study indicate that GSPs inhibit migration and invasion of bladder cancer cells by reversing EMT through suppression of the TGF-*β* signaling pathway. Our data suggest that GSPs may be developed as a potential chemopreventive and therapeutic agent against bladder cancer.

## Data Availability

The data used to support the findings of this study are included within the article.
